# Rationale, design, and methods: A randomized placebo-controlled trial of an immunomodulatory probiotic intervention for Veterans with PTSD

**DOI:** 10.1016/j.conctc.2022.100960

**Published:** 2022-06-28

**Authors:** Lisa A. Brenner, Kelly A. Stearns-Yoder, Christopher E. Stamper, Andrew J. Hoisington, Diana P. Brostow, Claire A. Hoffmire, Jeri E. Forster, Meghan L. Donovan, Arthur T. Ryan, Teodor T. Postolache, Christopher A. Lowry

**Affiliations:** a VA Rocky Mountain Mental Illness Research Education and Clinical Center (MIRECC), Rocky Mountain Regional VA Medical Center (RMRVAMC), Aurora, CO, United States; b Department of Physical Medicine & Rehabilitation, University of Colorado Anschutz Medical Campus, Aurora, CO, United States; c Departments of Psychiatry and Neurology, University of Colorado Anschutz Medical Campus, Aurora, CO, United States; d Military and Veteran Microbiome: Consortium for Research and Education, Aurora, CO, United States; e Department of Systems Engineering & Management, Air Force Institute of Technology, Wright-Patterson AFB, OH, United States; f Department of Psychiatry, University of Colorado Anschutz Medical Campus, Aurora, CO, United States; g Mood and Anxiety Program, University of Maryland School of Medicine, Baltimore, MD, United States; h VISN 5 MIRECC, Department of Veterans Affairs, Baltimore, MD, United States; i Department of Integrative Physiology, University of Colorado Boulder, Boulder, CO, United States; j Center for Neuroscience, University of Colorado Boulder, Boulder, CO, United States; k Center for Neuroscience, University of Colorado Anschutz Medical Campus, Aurora, CO, United States

**Keywords:** PTSD, Probiotics, Immunomodulation, Inflammation, Veterans, Randomized controlled trial

## Abstract

**Background:**

United States military Veterans from recent conflicts are experiencing symptoms related to posttraumatic stress disorder (PTSD). Many Veterans are resistant to conventional health and mental health interventions (e.g., medication, psychotherapy). Alternative treatment approaches are needed. An underlying feature of PTSD is exaggerated inflammation, both peripherally and in the central nervous system. This inflammation is thought to play an important role in the vulnerability to, aggravation of, and persistence of PTSD symptoms. Therefore, an innovative intervention strategy would be the use of immunoregulatory/anti-inflammatory probiotics to reduce inflammation. Here we describe the rationale, design, and methods of a randomized placebo-controlled trial (RCT) of *Lactobacillus rhamnosus* GG (LGG; ATCC 53103) for posttraumatic stress disorder (PTSD).

**Methods:**

This is a Phase IIa trial of LGG for United States military Veterans with PTSD, using a longitudinal, double-blind, randomized placebo-controlled design. The primary outcome measure is plasma concentration of high-sensitivity C-reactive protein.

**Conclusion:**

Despite the fact that symptoms associated with PTSD can be disabling, individuals living with this trauma-related disorder have limited options in terms of evidence-based interventions. Recent research efforts aimed at highlighting the biological mechanisms of PTSD suggest that increased inflammation and altered autonomic nervous system activity may be treatment targets, and that immunoregulatory probiotics, such as LGG, have the potential to decrease trauma-induced inflammatory responses, as well as associated symptoms. This manuscript describes the best powered human subjects Phase IIa trial, to date, of a probiotic intervention for those living with PTSD.

## Highlights

• The prevalence of trauma-related disorders is increasing, and interventions are limited.
• Inflammation and altered autonomic system activity may be treatment targets.
• Immunoregulatory probiotics may decrease inflammation and associated symptoms.
• This is a Phase IIa trial of LGG for United States military Veterans with PTSD.
• We are using a longitudinal, double-blind, randomized placebo-controlled design.

## 1 Introduction

Since October of 2001, approximately 2.7 million troops have been deployed in the recent conflicts, with many reporting mental health concerns upon return [1,2]. Posttraumatic stress disorder (PTSD), a trauma-related disorder that can result from exposure to an event that poses actual or threatened death or injury, has become increasingly prevalent. Although the estimated lifetime prevalence of PTSD in the United States (US) adult population is approximately 8.7% [3], findings suggest that approximately 20% of returning Service Members meet criteria for PTSD or associated mental health conditions [4]. Chronic PTSD symptoms associated with disruptions in the regulation of emotional and cognitive processes, including hypervigilance, low frustration tolerance/irritability, and impaired decision-making [5], can pose significant challenges for Service Members and Veterans*.* Thus, there is an ever-increasing need for the identification of new treatment options that are implementable, safe, and non-stigmatizing.

Treatment guidelines for PTSD commonly center around forms of psychotherapy, such as exposure-based or cognitive restructuring interventions [6]. However, traditional treatments can be ineffective, with non-response rates in outcome studies often reaching as high as 50% [7]. Further, many Operation Enduring Freedom/Operation Iraqi Freedom/Operation New Dawn (OEF/OIF/OND) Veterans can be resistant to engaging in conventional mental health treatments [8], highlighting the importance of exploring alternative interventions. Use of complementary and alternative medicine (CAM), particularly for symptoms associated with stress/trauma, has quickly risen in the US. Interestingly, research suggests that Veterans with PTSD may be more accepting of CAM approaches than those without this condition [9]. Taken together, these data indicate the need to explore novel CAM intervention strategies that can target mechanisms underlying PTSD symptomology.

Recent research efforts aimed at elucidating potential biological mechanisms of PTSD have facilitated the development of such novel interventions. Psychological trauma is known to be associated with altered autonomic nervous system activity [10] and impaired regulation of the hypothalamic–pituitary–adrenal (HPA) axis and insufficient glucocorticoid-signaling [11,12]. Evidence for inflammatory drivers of trauma-related disorders has also been accumulating [13,14]. For example, increased circulating levels of interleukin (IL)-6 immediately following trauma exposure have been found to predict the later development of PTSD symptoms [15]. Although not confirmed in all studies, low-grade inflammation has been shown to be associated with PTSD and physiological stress responses, as indicated by elevated serum C-reactive protein (CRP), IL-1β, IL-6, IL-12, interferon gamma (INFγ) and tumor necrosis factor (TNF) [[16], [17], [18], [19], [20], [21], [22], [23], [24]]. In prospective studies, high baseline plasma concentrations of CRP, measured pre-deployment among Military personnel, were associated with an increased likelihood of having PTSD symptoms post-deployment, suggesting that inflammation prior to trauma exposure may predispose individuals to developing a trauma-related condition [25]. In line with this finding, machine learning approaches have demonstrated that among features assessed prior to deployment, biomarkers of inflammation, including CRP, were among the highest-ranking features predicting PTSD symptoms following deployment [26]. Gene expression profiles of peripheral blood mononuclear cells (PBMCs) 1- and 4-months following trauma distinguish trauma survivors with PTSD from those without PTSD, signifying that altered immune functional status following trauma exposure is also predictive of PTSD [18]. Moreover, individuals with PTSD show enhanced spontaneous secretion of IL-1β, IL-6, and TNF from PBMCs, which correlate with symptom severity [18,27]. Anti-inflammatory interventions have been successfully used among those with other psychiatric disorders [[28], [29], [30], [31]]. Therefore, it is reasonable to suggest that anti-inflammatory or immunoregulatory agents may be a useful intervention for treating symptoms of PTSD.

The use of probiotics, or microorganisms deemed beneficial for human health, as a method for reducing inflammation has become a growing area of interest. *Lactobacillus rhamnosus* GG (LGG) is a gram-positive strain of the *Lactobacillus rhamnosus* species that is found in the human gut microbiome. LGG is immunoregulatory, increasing peripheral regulatory T cells (Treg) and anti-inflammatory cytokines including IL-10, and decreasing proinflammatory cytokines such as IL-6 [32,33]. It is one of the most widely used probiotic strains [34] and has been studied in relation to gastrointestinal health, stimulation of the immune response to improve vaccination efficacy, and prevention of allergic symptoms [[35], [36], [37], [38], [39]].

Accumulating evidence also suggests that probiotics with anti-inflammatory and immunoregulatory properties, such as LGG [32,33], have the potential to decrease trauma-induced inflammatory responses, while being highly accessible, low-cost, self-sustaining (e.g., portable) and, based on previous safety and tolerability trials, without significant side effects [40]. Moreover, in our recent pilot randomized placebo controlled trial among OEF/OIF Veterans with PTSD and mild traumatic brain injury (mTBI), supplementation with *Lactobacillus reuteri DSM 17938*, another probiotic with anti-inflammatory and immunoregulatory properties, was found to be feasible, acceptable, and safe [40], and was associated with decreases in plasma CRP that approached statistical significance, as well as decreases in an objective measure of distress (stress-induced increase in heart beats per minute [BPM]) during the Trier Social Stress Test) [40], a biomarker of autonomic stress responsivity [41,42].

**Fig. 1:**
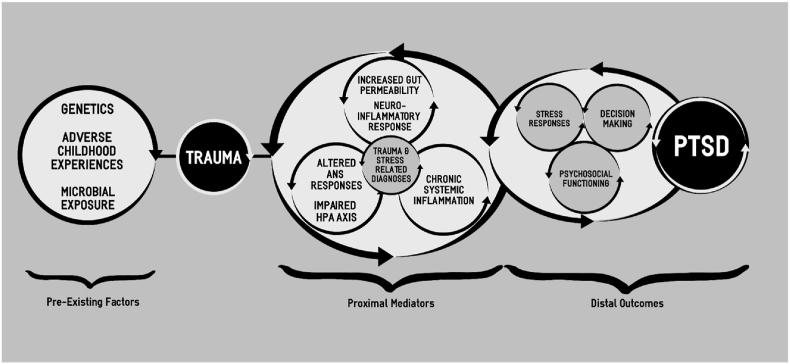
Working model of PTSD with an emphasis on immunomodulatory factors in the etiology and pathophysiology of PTSD. The model includes the influence of pre-existing factors that contribute to individual variability in physiological and psychological responses to trauma, leading to development and persistence of PTSD in a subset of individuals. Abbreviations: ANS, autonomic nervous system; HPA, hypothalamic-pituitary-adrenal; PTSD, posttraumatic stress disorder.

Based on previous research [14,22,23,43], we created a working model of PTSD, with an emphasis on immunomodulatory factors (see Fig. 1). The presented clinical trial is based on our overarching theses that: 1) elevated inflammation increases risk of developing PTSD; 2) chronic inflammation and impaired immunomodulation perpetuate symptoms among those with PTSD; 3) exacerbation of inflammation is associated with increased severity of PTSD symptoms; and, 4) administration of an anti-inflammatory/immunoregulatory probiotic is likely to influence an individual's gut microbial community, and decrease intestinal permeability (IP) and systemic inflammation, as well as dampen autonomic responsivity and PTSD symptoms.

The goal of this Phase IIa placebo-controlled randomized controlled trial (ClinicalTrials.gov Identifier: NCT04150380) is to investigate the effects of daily oral administration of LGG (ATCC 53103) in a cohort of OEF/OIF/OND Veterans with PTSD as assessed by the gold standard Clinician Administered PTSD Scale-5 (CAPS-5) [44] who also have Functional Bowel Disorders (FBD), including irritable bowel syndrome. Outcomes include plasma concentration of high-sensitivity CRP (mechanistic, primary), and PTSD symptom severity (clinical, exploratory) [45]. Additional biological signatures will be considered exploratory. The primary hypothesis is that those who receive LGG supplementation will respond with lower plasma levels of CRP as compared to those allocated to a placebo supplement. Exploratory hypotheses include, as compared to those allocated to placebo, those who receive LGG supplementation will respond with: 1) decreased PTSD symptoms; 2) increased abundance of LGG and community-level shifts (e.g., increased alpha diversity) in the gut microbiota (measured using RT-qPCR and DNA sequencing of the 16S rRNA gene, respectively), decreases in IP (decreased fatty acid binding protein 2 [FABP2] and D-amino acid oxidase [DAO]), increases in plasma concentrations of anti-inflammatory biomarkers (IL-10, IL-4), decreases in additional plasma biomarkers of inflammation (IL-6, IL-8, IFNγ, IL-1α, IL-1β, and IL-12p70), reduced stress responsivity (biological and psychological) during and after participation in the Cyberball task, and improved decision-making (measured by performance on the modified Iowa Gambling Test [mIGT]); and, 3) the effect of LGG supplementation on stress responsivity, decision-making, and PTSD symptom severity will be mediated by effects of LGG supplementation on the gut microbiota, IP, and plasma biomarkers of inflammation.

## 2 Materials and methods

### 2.1 Study design

**Fig. 2:**
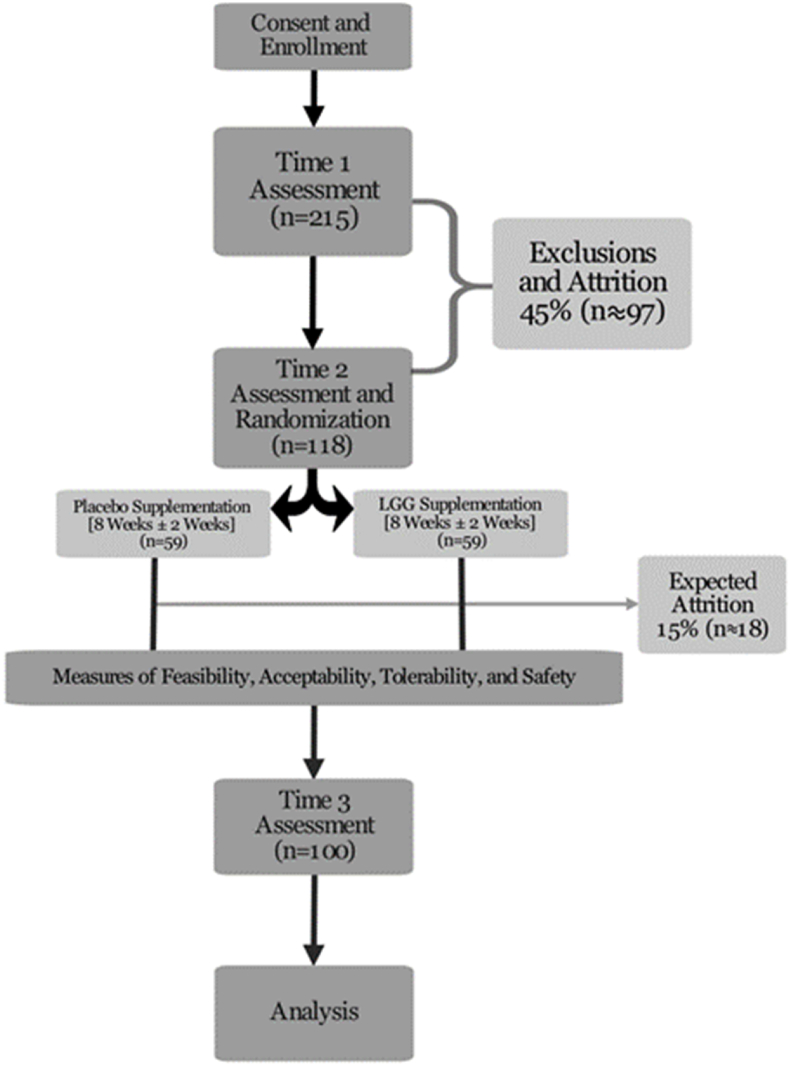
Consort diagram. Abbreviations: LGG, *Lactobacillus rhamnosus* GG.

The described Phase IIa trial will examine the effects of supplementation with LGG, a probiotic with anti-inflammatory and immunoregulatory properties, using a longitudinal, double-blind, randomized placebo-controlled design. It is expected that up to 215 Veterans will participate in initial evaluation procedures, with 118 being randomized to one of two interventions (probiotic or placebo supplementation; see Consort Diagram, Fig. 2). Randomization will be stratified by sex. This study is being conducted according to the guidelines outlined in the Declaration of Helsinki and all procedures involving human participants were approved by the Colorado Multiple Institutional Review Board (COMIRB).

**Fig. 3:**
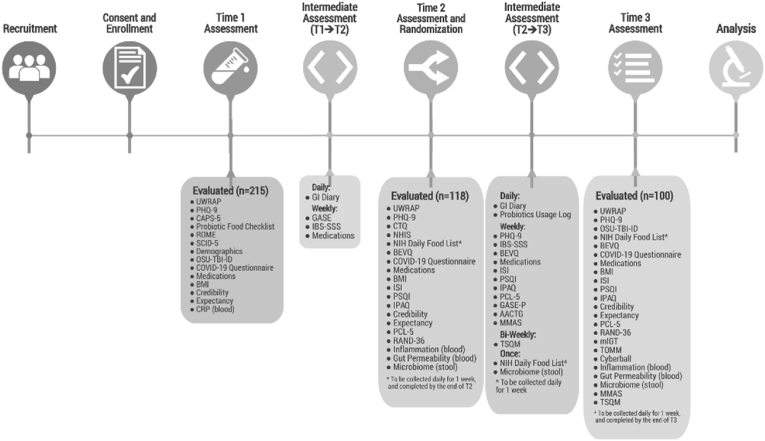
Participant flow diagram. Abbreviations: AACTG, Adult AIDS Clinical Trials Group; BEVQ, Beverage Intake Questionnaire (BEVQ-15); BMI, body mass index; CAPS-5, Clinician Administered PTSD Scale-5; COVID-19, Coronavirus Disease 2019; CRP, C-reactive protein; CTQ, Childhood Trauma Questionnaire; GASE, Generic Assessment of Side Effects; GASE-P, Generic Assessment of Side Effects – Probiotics; GI, gastrointestinal; IBS-SSS, Irritable Bowel Syndrome Severity Scoring System; IPAQ, International Physical Activity Questionnaire; ISI, Insomnia Severity Index; mIGT, Modified Iowa Gambling Task; MMIS, Modified Morisky Medication-Taking Adherence Scale; NHIS, National Health Interview Survey (NHIS) - Chronic Conditions; NIH, National Institutes of Health; OSU-TBI-ID, Ohio State University Traumatic Brain Injury (TBI) Identification Method; PCL-5, Posttraumatic Stress Disorder Checklist for DSM-5; PHQ-9, Patient Health Questionnaire-9; PSQI, Pittsburgh Sleep Quality Index; RAND-36, 36-Item Short Form Survey (SF-36) - RAND Corporation; ROME, Rome IV Diagnostic Questionnaire for the Adult Functional GI Disorders; SCID-5, Structured Clinical Interview for DSM-5-Research Version (SCID-5 for DSM-5, Research Version; SCID-5-RV, Version 1.0.0); T1, Time 1 assessment; T2, Time 2 assessment; T3, Time 3 assessment; TOMM, Test of Memory and Malingering; TSQM, Modified Treatment Satisfaction Questionnaire; UWRAP, University of Washington Risk Assessment Protocol-Revised.

At Time 1 (T1, Initial Study Assessment), diagnoses of PTSD and functional bowel disorder (FBD) will be confirmed. A blood draw will be conducted to identify an individual's baseline CRP level for inclusion, based on plasma CRP concentration >1.0 mg/L, the clinical threshold for average risk of cardiovascular disease [46]. Between Time 1 and Time 2 (T2, Assessment and Randomization Visit), participants will keep a pre-supplementation daily diary of gastrointestinal (GI) symptoms for approximately 2 weeks. At T2, data will bei collected regarding group profile/potential confounding variable measures, psychological outcomes, and biological signatures. Between T2 and Time 3 (T3, End of Study Period Assessment), individuals will supplement with LGG or placebo (for 8±2 weeks), and measures of tolerability, safety, acceptability, and feasibility will be collected. Data regarding diet, sleep, exercise, and PTSD symptoms will also be collected (via phone or email surveys). At T3, data will be collected regarding psychological outcomes (e.g., PTSD symptoms), and biological signatures (e.g., inflammation), as well as potential covariates (see Fig. 3, Participant Flow Diagram).

### 2.2 Probiotic and placebo interventions

LGG ATCC strain 53103 (Chr. Hansen A/S, 1.0 x 10^10^ colony-forming units (CFUs)/capsule) is being used in the proposed study. Participants will be randomized to one of two conditions in which they are instructed to consume one LGG or placebo supplementation capsule daily, for 8±2 weeks.

Through traditional and molecular methods, our team will conduct independent testing regarding the purity and viability of the probiotic upon receipt of the capsules and after the expected shelf life. After initial receipt, a randomized subsampling of 10 capsules (∼0.5%) will be analyzed, and another 10 pills will be stored in the lab under the same conditions as recommended to the participants in the study. An identical process will be conducted for placebo pills to verify absence of LGG. The viability and CFUs of the probiotic and placebo will be confirmed against the manufacturer's documentation by culturing on *Lactobacillus* selection agar plates. Furthermore, the purity and quantity of the probiotic will be confirmed against the manufacturer's documentation by reverse transcription quantitative polymerase chain reaction (RT-qPCR).

### 2.3 Study population

Potential participants will be willing and eligible US military Veterans between the ages of 18 and 60 from the following populations: 1) those seeking outpatient mental health, rehabilitative, psychological, or other services within a VA Health Care System; 2) those in existing clinical and research databases; and 3) Veterans in the community not seeking care at the VA. Inclusion and exclusion criteria are presented in Table 1.

**Table 1:** Inclusion and exclusion criteria.

**Inclusion criteria**
History of at least one deployment in support of Operation Enduring Freedom/Operation Iraqi Freedom/Operation New Dawn (OEF/OIF/OND)
Current diagnosis of PTSD per the Clinician Administered PTSD Scale-5 (CAPS-5)
Current diagnosis of a Functional Bowel Disorder including Irritable Bowel Syndrome [IBS]) by the ROME IV
CRP level of 1.0 mg/L or above at Time 1
Medical clearance to participate by study providers
Age between 18 and 60
Ability to provide informed consent
Willingness to abstain from probiotic supplements (pills, tablets, oils, foods, etc.) other than the investigational product provided until all study procedures are completed
Willingness to provide blood and stool samples
**Exclusion criteria**
Inability to adequately respond to questions regarding the informed consent procedure
Currently involved in the criminal justice system as a prisoner or ward of the state
Non-English speaking
Current (past month) alcohol or substance abuse or dependence, excluding marijuana
Lifetime history of bipolar disorder or psychosis or anxiety disorders
Consistent (e.g., 5x/week or greater) probiotic supplementation within the last month, including probiotic food products such as yogurt, as determined by phone screen interview and Probiotic Food Check List
Receiving intravenous, intramuscular, systemic or oral antibiotics within the last month
Presence of central venous catheters (CVCs)
Gastrointestinal (GI) barriers as identified by the 2-week run-in period as determined by the study team
Participation in conflicting interventional research protocol
Vital signs outside of acceptable range, i.e., blood pressure >160/100, pulse >100
Use of any of the following drugs within the last 6 months: antifungals, antivirals or antiparasitics (intravenous, intramuscular, or oral); oral, intravenous, intramuscular, or inhaled corticosteroids; cytokines or cytokine inhibitors; methotrexate or immunosuppressive cytotoxic agents
Acute disease at the time of enrollment (defer sampling until the participant recovers). Acute disease is defined as the presence of a moderate or severe illness with or without fever (e.g., oral temperature >100 °F)
Any medical condition deemed exclusionary by the Principal Investigators
History of cancer
Unstable dietary history as determined by the PIs (e.g., major changes in diet during the previous month, where the subject has eliminated or significantly increased a major food group in the diet)
Positive test for human immunodeficiency virus (HIV), Hepatitis B virus, or Hepatitis C virus
Any confirmed or suspected condition/state of immunosuppression or immunodeficiency (primary or acquired) including HIV infection, or those receiving immunosuppressive drugs or treatment including antineoplastic therapy, post-transplantation immunosuppressive therapy, and/or radiation therapy
Major surgery of the GI tract, with the exception of cholecystectomy and appendectomy, in the past five years. Any major bowel resection at any time
Female who is pregnant or lactating
Treatment for or suspicion of ever having had toxic shock syndrome
History of moderate and/or severe traumatic brain injury

### 2.4 Measures and variable definitions

For a full list of measures and variable definitions, see Table 2.

**Table 2:** Study measures/outcomes and variable definitions.

Measure	Condition(s)/Factor(s) of Interest
Adult AIDS Clinical Trials Group (AACTG)	Supplement adherence
Beverage Intake Questionnaire (BEVQ-15)	Frequency/quantity of intake of beverages
Bristol Stool Scale	Number of daily bowel movements and consistency of stool
C-reactive protein (CRP)^a^	Inflammation
Clinician Administered PTSD Scale-5 (CAPS-5)	Current PTSD diagnosis
Childhood Trauma Questionnaire (CTQ)	Childhood abuse experiences
COVID-19 Questionnaire	Impact of COVID-19 pandemic and stressors related to the pandemic
Credibility Scale	Sense of supplementation credibility
D-amino acid oxidase (DAO)^b^	Intestinal permeability
Expectancy Scale	Expectations regarding supplementation
Fatty acid binding protein 2 (FABP2)^b^	Intestinal permeability
Generic Assessment of Side Effects (GASE)	Medication side effects
Generic Assessment of Side Effects – Probiotics (GASE-P)	Supplement side effects
Heart rate variability (HRV)	Physiological stress
Height and weight	Body Mass Index (BMI)
Interleukin (IL)-6, interferon gamma (IFNγ), tumor necrosis factor (TNF), IL-1α, IL-1β, IL-12p70, IL-8, IL-4, and IL-10^b^	Inflammation
Insomnia Severity Index (ISI)	Insomnia symptom severity
International Physical Activity Questionnaire (IPAQ)	Physical activity
Irritable Bowel Syndrome Severity Scoring System (IBS-SSS)	Severity and frequency of abdominal pain and abdominal distension, dissatisfaction with bowel habits, and interference with quality of life
Medications Log	Current medications and dosage
Modified Iowa Gambling Task (mIGT)	Decision making
Modified Morisky Medication-Taking Adherence Scale (MMAS)	Supplement adherence
Modified Treatment Satisfaction Questionnaire (TSQM v1.4)	Supplement side effects
National Health Interview Survey (NHIS) - Chronic Conditions	Chronic health symptoms
National Institutes of Health Daily Food List	Food intake
Ohio State University Traumatic Brain Injury (TBI) Identification Method (OSU TBI-ID)	Lifetime history of TBI
Patient Health Questionnaire-9	Depression symptom severity
Pittsburgh Sleep Quality Index	Subjective sleep duration
Posttraumatic Stress Disorder Checklist for DSM-5 (PCL-5)^b^	PTSD symptom severity
Probiotic Food Check List	Consumption of probiotic-containing food products
Psychological Stress Measure (PSM)^b^	Perceived stress
Rocky Mountain MIRECC Demographic Form	Demographics
Rome IV Diagnostic Questionnaire for the Adult Functional GI Disorders	Digestive system disorders
Structured Clinical Interview for DSM-5-Research Version (SCID-5 for DSM-5, Research Version; SCID-5-RV, Version 1.0.0)	Axis I psychiatric disorders
Supplement Usage Log	Date, time, and amount of each probiotic dose
Test of Memory and Malingering (TOMM)	Memory
University of Washington Risk Assessment Protocol-Revised (UWRAP)	Risk assessment
Veterans RAND 36, 36-Item Short Form Survey (SF-36) - RAND Corporation	Health-related quality of life
Visual Analog Scale (VAS)^b^	Perceived stress

a Primary outcome.

b Additional outcomes measures.

### 2.5 Outcome measures

The primary mechanistic outcome for this study is plasma concentration of high-sensitivity CRP. Exploratory outcomes include: 1) PTSD symptom severity (PTSD Symptom Checklist for DSM-5; PCL-5) [47]; 2) type and abundance of LGG and community-level gut microbiota (measured using qRT-PCR and DNA sequencing of the 16S rRNA gene); 3) IP (FABP2 and DAO; 4) plasma concentrations of IL-10, IL-4, IL-6, IL-8, IFNγ, IL-1α, IL-1β, and IL-12; 5); biological and psychological stress responsivity during and after participation in the Cyberball task [48]; and, 6) decision-making (mIGT). All outcomes are listed in Table 2.

### 2.6 Safety monitoring

Adverse Events from the Study Drug. Participants will be asked about GI symptoms throughout the study as identified in procedures. Participants will be asked about new symptoms, adverse events and any new medications or supplements during the weekly check-in phone call using the Generic Assessment of Side Effects (GASE)/Generic Assessment of Side Effects – Probiotics (GASE-P).

Suicide Risk Assessment and Management. All research staff will also complete Veterans Affairs (VA) required research trainings. Study staff will contact the PI or her clinical designee if other concerning information regarding the participant's safety or the safety of others is identified during administration of the protocol. Assessors will have emergency contact information available at all times for the PI or designee, as well as crisis management and emergency services (e.g., VA National Veterans Crisis Line, Psychiatric Emergency Services, 911). If contacted, the PI or her clinical designee will conduct a more thorough risk evaluation and will initiate appropriate follow-up procedures.

For assessments conducted over the phone, when indicated, assessors will stay on the telephone with the participant until they are able to consult with the PI or clinical designee, determine next steps, and follow through with them. Prior to beginning the phone assessment, assessors will ask participants to provide the address of the location they are at while completing the phone assessment so that the assessor, after consulting with the PI or designated clinician, can direct emergency services to the participant's location when warranted. With respect to confidentiality, prior to participating in the study, informed consent documents will contain statements explaining mandatory reporting requirements for information regarding intention to harm self or others. In situations where confidentiality is breached due to threats to participants' or others' safety (e.g., suicide or homicide risk), precautions will be taken to protect their privacy by limiting the information being disclosed to only the minimum information necessary to ensure the safety and welfare of participants and others affected.

Adverse Events. Significant adverse events are not expected. For this study, adverse events are defined as symptoms reported by the participant that are directly related to participation in the study. The study PIs will be notified of any complaints or reports of adverse events. Data and safety monitoring will be regularly reviewed by the study personnel and the Study PIs to ensure protocol adherence and participant safety. Any unexpected serious adverse events (uSAEs), defined as death, life threatening illness, suicide attempt, hospitalization or prolonged hospitalization, and persistent/significant disability, will be reported to COMIRB by the study PIs within 5 days of them being made aware. Other adverse events will be recorded and reported at continuing review. In addition, there is a Data Safety Monitor Board (DSMB) who will review data produced by the study team and will meet with the lead investigators and study statistician on a regular basis.

### 2.7 Study modifications during COVID-19 pandemic

Given challenges associated with in-person research due to the COVID-19 pandemic, the PIs made approved study modifications that allowed for continuance of the trial. Specifically, we modified T3 to include remote strategies both in terms of the stressor and strategy for measuring heart rate variability (HRV). This included switching from our original stressor, the Trier Social Stress Test [49] to Cyberball [48], which is a virtual ball-toss game that can be used for research on ostracism, social exclusion, or rejection. During Cyberball, the participant is told that they are playing a computerized ball tossing game with three other players over the internet: in reality, the other ‘players’ characters are controlled by the computer. After a few initial trials where the other players toss the ball to the participant, the other players then exclude the participant, only throwing to one another. This exclusionary experience has been found to be distressing to participants [50].

For HRV measurement, we will use the Firstbeat Bodyguard 2 (BG2) [51]. BG2 is a beat-to-beat heart rate monitoring device that is intended for short-term or long-term monitoring of heart rate variability and physical activity. The device records ECG with electrodes, processes the signal with an integrated algorithm and provides beat-to-beat R-to-R interval as an output with 1 ms resolution. The device easily attaches directly to the skin with two chest electrodes and starts recording data automatically. The BG2 has been designed for 24 h recordings. R-to-R timing data is downloaded from the BG2 to a computer for further analysis using standard HRV analysis software.

### 2.8 Data analysis

All analyses will use a two-sided test of hypotheses, with a significance level of 0.05 unless otherwise noted, and will be run in SAS v9.4 or higher, R v4.1.1 or higher, or QIIME2 2019.1 or higher. Group profile variables will be summarized as means and standard deviations (SD), medians and ranges, or *N* and percent and will be compared between those allocated to LGG supplementation and those allocated to placebo supplementation using *t*-tests, Wilcoxon rank-sum tests, chi-square tests and Fisher's exact tests, as appropriate. Group profile variables that are found to be significant at the *p* < 0.10 level and are considered as plausible potential confounders will be included as noted in the analysis plans below, although given randomization, the number meeting this criterion will be low. These may include demographics, history of TBI, childhood trauma, chronic health conditions, diet, beverage consumption, medications, BMI, insomnia severity, sleep duration and quality, and/or exercise. Data from the Test of Memory and Malingering (TOMM) [52,53] will be used to increase understanding regarding participants' ability to engage in mIGT testing. mIGT scores will not be used from those whose TOMM scores suggest poor effort/engagement.

The recruitment goal for this study is 59 participants per group. Anticipating a 15% attrition rate, we will have 50 participants per group for the final analysis. For the primary outcome of plasma concentration of CRP inflammatory biomarker, linear regression will be used to model the changes between T2 and T3, as a function of group, sex, the baseline value of CRP and any potential confounders identified as noted above. A similar analysis will be performed for the exploratory clinical outcome of PTSD symptoms (PCL-5 score). For the gut microbiome, linear regression will be used to model the changes between T2 and T3 both in alpha diversity, measured by Shannon Diversity Index, and in the gut microbiota community, quantified by Unweighted and Weighted UniFrac [54], pairwise distance between times for each individual, as a function of group, sex, the baseline value of the outcome, and potential confounders. Linear regression will also be used to model changes between T2 and T3 in IP (fatty acid binding protein 2 [FABP2], D-amino acid oxidase [DAO]), as well as plasma biomarkers of inflammation (IL-6, IL-10, IFNγ, IL-1α, IL-1β, IL-12p70, IL-8, and IL-4), and decision making as a function of group, sex, the baseline value of the outcome, and potential confounders. For stress outcomes from Cyberball (HRV, visual analog scale [VAS] and psychological stress measure [PSM]), linear regression will be used to model each outcome at T3 as a function of group. For each outcome measured at T2 and T3, the estimated mean difference in change between the groups will be reported with 95% confidence intervals (CI). For the stress outcomes measured only at T3 the estimated mean differences between the groups at T3 will be reported with 95% CIs. For the mediation analysis, we will use methods proposed by VanderWeele et al. for multiple mediators [55,56], examining whether the relationships between probiotic treatment and the outcome measures (PTSD symptoms, stress response, decision making) are mediated by changes in the gut microbiome, IP, and inflammation.

Assuming a CRP standard deviation of 1.19 mg/L as observed in Brenner et al. [40], 50 participants per group provides 85% power to detect a difference in change between groups of 0.73 mg/L at the α = 0.05 level for the primary outcome.

## 3 Discussion

Military personnel's recent exposures in Iraq and Afghanistan have resulted in a new generation of Veterans with persistent symptoms of PTSD. Moreover, stressors associated with the COVID-19 pandemic have placed both patients [57] and health care providers [58] at risk for developing trauma-related disorders. As such, novel means of intervention are required. In particular, efficacious interventions that are portable, non-stigmatizing, and cost-effective, such as probiotics, would be welcome.

As highlighted above, nonresponse rates in outcomes studies of PTSD have been notably high [7]. Schottenenbauer et al. explored possible predictors of nonresponse and dropout and highlighted the writing of Hembree and Foa, which suggested that those who need therapy the most have the greatest likelihood of dropping out or not responding to care [59]. In response, some have suggested that a subset of individuals may benefit from pre-treatment (e.g., skills training) to decrease dropout. However, Hembree and Foa also noted that dropout appears to be at least in part related to the complexity of treatment [60]. An alternate means of addressing this challenge would be to introduce an intervention, such as probiotics, that could alter patterns of autonomic activity during exposure to stressful experiences [40]. This strategy may facilitate sustained treatment engagement, even during periods in which the content of material being discussed during sessions is distressing.

Moreover, efforts outlined as part of this clinical trial are aimed at increasing understanding regarding inflammation, the gut microbiome, autonomic functioning (i.e., HRV), and PTSD symptoms. Such knowledge is expected to highlight multiple potential interventions for PTSD, as well as other mental health-related conditions that involve inappropriate inflammation and dysregulated autonomic function, for example long-COVID [61]. Also of note, Reber et al. recently completed an animal study in which mice were treated with a heat-killed preparation of an immunoregulatory environmental microorganism, *Mycobacterium vaccae* NCTC 11659 [62]. Findings showed that animals that were treated with *M. vaccae* NCTC 11659 were significantly more resistant to stress-induced pathology, thereby suggesting that immunoregulatory microorganisms may also have a role in preventing trauma-based disorders [62].

## 4 Conclusions

In summary, the study outlined above is expected to provide low risk of bias data regarding the effects of LGG on multiple outcomes, including inflammation and PTSD symptoms. Given the increasing rate of PTSD, as well as current limitations of efficacious and frequently implemented treatments, these results are expected to provide evidence to inform alternate means of addressing the needs of the wide range of individuals living with or at risk for developing trauma-related conditions.

## Funding

This work was supported by the 10.13039/100000002National Institutes of Health, 10.13039/100008460National Center for Complementary and Integrative Health [Grant Number 5R01AT010005-02]; The writing of this manuscript was also supported by the Office of Academic Affiliations, Advanced Fellowship Program in Mental Illness Research and Treatment, Department of Veterans Affairs.

## Declaration of interest

Dr. Lowry serves on the Scientific Advisory Board of Immodulon Therapeutics Ltd. and is a member of the faculty of the Integrative Psychiatry Institute, Boulder, Colorado. Dr. Brenner reports grants from the VA, DOD, NIH, and the State of Colorado, editorial renumeration from Wolters Kluwer, and royalties from the American Psychological Association and Oxford University Press. In addition, she consults with sports leagues via her university affiliation.

## Data Availability

No data were used for the research described in the article.

## References

[1] Wenger J.W., O'Connell C., Cottrell L. (2018). https://apps.dtic.mil/sti/pdfs/AD1085604.pdf.

[2] Hoge C.W., Auchterlonie J.L., Milliken C.S. (2006). Mental health problems, use of mental health services, and attrition from military service after returning from deployment to Iraq or Afghanistan. JAMA.

[3] American Psychiatric Association (2013). Diagnostic and Statistical Manual of Mental Disorders.

[4] Schlenger W.E., Kulka R.A., Fairbank J.A. (1992). The prevalence of post‐traumatic stress disorder in the Vietnam generation: a multimethod, multisource assessment of psychiatric disorder. J. Trauma Stress.

[5] Tanielian T., Haycox L.H., Schell T.L. (2008). https://www.rand.org/content/dam/rand/pubs/monographs/2008/RAND_MG720.1.pdf.

[6] (2010). VA/DoD Clinical Practice Guideline for Management of Post-traumatic Stress. Version 2.0.

[7] Schottenbauer M.A., Glass C.R., Arnkoff D.B., Tendick V., Gray S.H. (2008). Nonresponse and dropout rates in outcome studies on PTSD: review and methodological considerations. Psychiatry.

[8] Kim P.Y., Thomas J.L., Wilk J.E., Castro C.A., Hoge C.W. (2010). Stigma, barriers to care, and use of mental health services among active duty and National Guard soldiers after combat. Psychiatr. Serv..

[9] Betthauser L.M., Brenner L.A., Forster J.E., Hostetter T.A., Schneider A.L., Hernández T.D. (2014). A factor analysis and exploration of attitudes and beliefs toward complementary and conventional medicine in veterans. Med. Care.

[10] Dedert E.A., Calhoun P.S., Watkins L.L., Sherwood A., Beckham J.C. (2010). Posttraumatic stress disorder, cardiovascular, and metabolic disease: a review of the evidence. Ann. Behav. Med..

[11] Dunlop B.W., Wong A. (2019). The hypothalamic-pituitary-adrenal axis in PTSD: pathophysiology and treatment interventions. Prog. Neuro-Psychopharmacol. Biol. Psychiatry.

[12] Szeszko P.R., Lehrner A., Yehuda R. (2018). Glucocorticoids and hippocampal structure and function in PTSD. Harv. Rev. Psychiatr..

[13] Neigh G.N., Ali F.F. (2016). Co-morbidity of PTSD and immune system dysfunction: opportunities for treatment. Curr. Opin. Pharmacol..

[14] Speer K., Upton D., Semple S., McKune A. (2018). Systemic low-grade inflammation in post-traumatic stress disorder: a systematic review. J. Inflamm. Res..

[15] Pervanidou P., Kolaitis G., Charitaki S. (2007). Elevated morning serum interleukin (IL)-6 or evening salivary cortisol concentrations predict posttraumatic stress disorder in children and adolescents six months after a motor vehicle accident. Psychoneuroendocrinology.

[16] Song Y., Zhou D., Guan Z., Wang X. (2007). Disturbance of serum interleukin-2 and interleukin-8 levels in posttraumatic and non-posttraumatic stress disorder earthquake survivors in northern China. Neuroimmunomodulation.

[17] von Känel R., Hepp U., Kraemer B. (2007). Evidence for low-grade systemic proinflammatory activity in patients with posttraumatic stress disorder. J. Psychiatr. Res..

[18] Gola H., Engler H., Sommershof A. (2013). Posttraumatic stress disorder is associated with an enhanced spontaneous production of pro-inflammatory cytokines by peripheral blood mononuclear cells. BMC Psychiatr..

[19] Lindqvist D., Wolkowitz O.M., Mellon S. (2014). Proinflammatory milieu in combat-related PTSD is independent of depression and early life stress. Brain Behav. Immun..

[20] Page M.J., Bester J., Pretorius E. (2018). Interleukin‐12 and its procoagulant effect on erythrocytes, platelets and fibrin (ogen): the lesser known side of inflammation. Br. J. Haematol..

[21] Zhou J., Nagarkatti P., Zhong Y. (2014). Dysregulation in microRNA expression is associated with alterations in immune functions in combat veterans with post-traumatic stress disorder. PLoS One.

[22] Spitzer C., Barnow S., Völzke H. (2010). Association of posttraumatic stress disorder with low-grade elevation of C-reactive protein: evidence from the general population. J. Psychiatr. Res..

[23] Plantinga L., Bremner J.D., Miller A.H. (2013). Association between posttraumatic stress disorder and inflammation: a twin study. Brain Behav. Immun..

[24] Baker D.G., Nievergelt C.M., O'Connor D.T. (2012). Biomarkers of PTSD: neuropeptides and immune signaling. Neuropharmacology.

[25] Eraly S.A., Nievergelt C.M., Maihofer A.X. (2014). Assessment of plasma C-reactive protein as a biomarker of posttraumatic stress disorder risk. JAMA Psychiatr..

[26] Schultebraucks K., Qian M., Abu-Amara D. (2020). Pre-deployment risk factors for PTSD in active-duty personnel deployed to Afghanistan: a machine-learning approach for analyzing multivariate predictors. Mol. Psychiatr..

[27] Andrews J.A., Neises K.D. (2012). Cells, biomarkers, and post‐traumatic stress disorder: evidence for peripheral involvement in a central disease. J. Neurochem..

[28] Miller A.H., Raison C.L. (2015). Are anti-inflammatory therapies viable treatments for psychiatric disorders?: where the rubber meets the road. JAMA Psychiatr..

[29] Raison C.L., Rutherford R.E., Woolwine B.J. (2013). A randomized controlled trial of the tumor necrosis factor antagonist infliximab for treatment-resistant depression: the role of baseline inflammatory biomarkers. JAMA Psychiatr..

[30] Müller N., Schwarz M., Dehning S. (2006). The cyclooxygenase-2 inhibitor celecoxib has therapeutic effects in major depression: results of a double-blind, randomized, placebo controlled, add-on pilot study to reboxetine. Mol. Psychiatr..

[31] Chaudhry I.B., Hallak J., Husain N. (2012). Minocycline benefits negative symptoms in early schizophrenia: a randomised double-blind placebo-controlled clinical trial in patients on standard treatment. J. Psychopharmacol..

[32] Khailova L., Baird C.H., Rush A.A., Barnes C., Wischmeyer P.E. (2017). *Lactobacillus rhamnosus* GG treatment improves intestinal permeability and modulates inflammatory response and homeostasis of spleen and colon in experimental model of pseudomonas aeruginosa pneumonia. Clin. Nutr..

[33] Chen R.-C., Xu L.-M., Du S.-J. (2016). *Lactobacillus rhamnosus* GG supernatant promotes intestinal barrier function, balances Treg and TH17 cells and ameliorates hepatic injury in a mouse model of chronic-binge alcohol feeding. Toxicol. Lett..

[34] Segers M.E., Lebeer S. (2014). Towards a better understanding of *Lactobacillus rhamnosus* GG-host interactions. Microb.

[35] Isolauri E., Joensuu J., Suomalainen H., Luomala M., Vesikari T. (1995). Improved immunogenicity of oral D x RRV reassortant rotavirus vaccine by *Lactobacillus casei* GG. Vaccine.

[36] de Vrese M., Rautenberg P., Laue C., Koopmans M., Herremans T., Schrezenmeir J. (2005). Probiotic bacteria stimulate virus–specific neutralizing antibodies following a booster polio vaccination. Eur. J. Nutr..

[37] Hojsak I., Abdović S., Szajewska H., Milošević M., Ž Krznarić, Kolaček S. (2010). *Lactobacillus* GG in the prevention of nosocomial gastrointestinal and respiratory tract infections. Pediatrics.

[38] Davidson L.E., Fiorino A.-M., Snydman D.R., Hibberd P.L. (2011). *Lactobacillus* GG as an immune adjuvant for live-attenuated influenza vaccine in healthy adults: a randomized double-blind placebo-controlled trial. Eur. J. Clin. Nutr..

[39] Pelto L., Isolauri E., Lilius E., Nuutila J., Salminen S. (1998). Probiotic bacteria down-regulate the milk-induced inflammatory response in milk-hypersensitive subjects but have an immunostimulatory effect in healthy subjects. Clin. Exp. Allergy.

[40] Brenner L.A., Forster J.E., Stearns-Yoder K.A. (2020). Evaluation of an immunomodulatory probiotic intervention for veterans with co-occurring mild traumatic brain injury and posttraumatic stress disorder: a pilot study. Front. Neurol..

[41] Kupper N., Jankovic M., Kop W.J. (2021). Individual differences in cross-system physiological activity at rest and in response to acute social stress. Psychosom. Med..

[42] Hellhammer J., Schubert M. (2012). The physiological response to Trier Social Stress Test relates to subjective measures of stress during but not before or after the test. Psychoneuroendocrinology.

[43] Heath N.M., Chesney S.A., Gerhart J.I. (2013). Interpersonal violence, PTSD, and inflammation: potential psychogenic pathways to higher C-reactive protein levels. Cytokine.

[44] U.S. Department of Veterans Affairs Clinician administered PTSD scale for DSM-5 (CAPS-5). https://www.ptsd.va.gov/professional/assessment/adult-int/caps.asp.

[45] Ng Q.X., Soh A.Y.S., Loke W., Venkatanarayanan N., Lim D.Y., Yeo W.S. (2019). Systematic review with meta‐analysis: the association between post‐traumatic stress disorder and irritable bowel syndrome. J. Gastroenterol. Hepatol..

[46] Myers G.L., Rifai N., Tracy R.P. (2004). CDC/AHA workshop on markers of inflammation and cardiovascular disease: application to clinical and public health practice: report from the laboratory science discussion group. Circulation.

[47] Weathers F.W., Litz B.T., Keane T.M., Palmieri P.A., Marx B.P., Schnurr P.P. (2013).

[48] Williams K.D., Jarvis B. (2006). Cyberball: a program for use in research on interpersonal ostracism and acceptance. Behav. Res. Methods.

[49] Kirschbaum C., Pirke K.-M., Hellhammer D.H. (1993). The ‘Trier Social Stress Test’–a tool for investigating psychobiological stress responses in a laboratory setting. Neuropsychobiology.

[50] Hartgerink C.H., Van Beest I., Wicherts J.M., Williams K.D. (2015). The ordinal effects of ostracism: a meta-analysis of 120 cyberball studies. PLoS One.

[51] Parak J., Korhonen I. (2013).

[52] Rees L.M., Tombaugh T.N., Gansler D.A., Moczynski N.P. (1998). Five validation experiments of the test of memory malingering (TOMM). Psychol. Assess..

[53] Tombaugh T.N. (1997). The Test of Memory Malingering (TOMM): normative data from cognitively intact and cognitively impaired individuals. Psychol. Assess..

[54] Lozupone C., Lladser M.E., Knights D., Stombaugh J., Knight R. (2011). Unifrac: an effective distance metric for microbial community comparison. ISME J..

[55] VanderWeele T.J. (2016). Mediation analysis: a practitioner's guide. Annu. Rev. Publ. Health.

[56] VanderWeele T., Vansteelandt S. (2014). Mediation analysis with multiple mediators. Epidemiol. Methods.

[57] Janiri D., Carfì A., Kotzalidis G.D. (2021). Posttraumatic stress disorder in patients after severe COVID-19 infection. JAMA Psychiatr..

[58] Vindegaard N., Benros M.E. (2020). COVID-19 pandemic and mental health consequences: systematic review of the current evidence. Brain Behav. Immun..

[59] Hembree E.A., Foa E.B. (2003). Interventions for trauma‐related emotional disturbances in adult victims of crime. J. Trauma Stress.

[60] Hembree E.A., Foa E.B., Dorfan N.M., Street G.P., Kowalski J., Tu X. (2003). Do patients drop out prematurely from exposure therapy for PTSD?. J. Trauma Stress.

[61] Tizenberg B.N., Brenner L.A., Lowry C.A. (2021). Biological and psychological factors determining neuropsychiatric outcomes in COVID-19. Curr. Psychiatr. Rep..

[62] Reber S.O., Siebler P.H., Donner N.C. (2016). Immunization with a heat-killed preparation of the environmental bacterium *Mycobacterium vaccae* promotes stress resilience in mice. Proc. Natl. Acad. Sci. USA.

